# Synergistic effects of SAP and PGPR on physiological characteristics of leaves and soil enzyme activities in the rhizosphere of poplar seedlings under drought stress

**DOI:** 10.3389/fpls.2024.1485362

**Published:** 2024-10-30

**Authors:** Dawei Jing, Fangchun Liu, Shanwen Li, Yufeng Dong

**Affiliations:** ^1^ College of Ecology, Resources and Environment, Dezhou University, Dezhou, China; ^2^ Institute of Resource and Environment, Shandong Academy of Forestry, Jinan, China; ^3^ Key Laboratory for Genetic Improvement in Forest Trees of Shandong Province, Shandong Academy of Forestry, Jinan, China; ^4^ Shandong Academy of Forestry, Jinan, China

**Keywords:** endogenous hormone balance, plant growth-promoting rhizobacteria, *populus* × *canadensis* ‘huaxiong 1’ seedling, rhizosphere soil enzyme, super absorbent polymers

## Abstract

Super absorbent polymers (SAP) provide moisture conditions that allow plant growth-promoting rhizobacteria (PGPR) to enter the soil for acclimatization and strain propagation. However, the effects of SAP co-applied with PGPR on the physiological characteristics of leaves and rhizosphere soil enzyme activities of poplar seedlings are not well understood. Here, a pot experiment using one-year-old poplar seedlings with five treatments, normal watering, drought stress (DR), drought stress + SAP (DR+SAP), drought stress + *Priestia megaterium* (DR +PGPR) and drought stress + SAP + *P. megaterium* (DR+S+P), was performed to analyze the contents of non-enzymatic antioxidants, osmotic regulators and hormones in leaves, as well as rhizosphere soil enzyme activities. Compared with normal watering, the DR treatment significantly decreased the contents of dehydroascorbate (DHA; 19.08%), reduced glutathione (GSH; 14.18%), oxidized glutathione, soluble protein (26.84%), indoleacetic acid (IAA; 9.47%), gibberellin (GA) and zeatin (ZT), the IAA/abscisic acid (ABA), GA/ABA, ZT/ABA and (IAA+GA+ZT)/ABA (34.67%) ratios in leaves, and the urease and sucrase activities in the rhizosphere soil. Additionally, it significantly increased the soluble sugar, proline and ABA contents in leaves. However, in comparison with the DR treatment, the DR+S+P treatment significantly increased the DHA (29.63%), GSH (15.13%), oxidized glutathione, soluble protein (29.15%), IAA (12.55%) and GA contents, the IAA/ABA, GA/ABA, ZT/ABA and (IAA+GA+ZT)/ABA (46.85%) ratios in leaves, and the urease, sucrose and catalase activities in rhizosphere soil to different degrees. The soluble sugar, proline and ABA contents markedly reduced in comparison to the DR treatment. The effects of the DR+SAP and DR+PGPR treatments were generally weaker than those of the DR+S+P treatment. Thus, under drought-stress conditions, the simultaneous addition of SAP and *P. megaterium* enhanced the drought adaptive capacities of poplar seedlings by regulating the non-enzymatic antioxidants, osmotic regulators, and endogenous hormone content and balance in poplar seedling leaves, as well as by improving the rhizosphere soil enzyme activities.

## 1 Introduction

Poplar is the main tree species used for establishing shelterbelts and timber forests in the northern plains and sandy areas of China. With a wide variety of species and a large cultivation area, poplar plays an irreplaceable role in water and soil conservation. Additionally, the development of poplar plantation forests is a major initiative to alleviate the global shortage of forest resources, to meet the needs of social development and to promote economic development (Štochlová et al., 2019). Also, poplar is considered an ideal tree species for carbon sink and bioenergy forests (Balasubramanian et al., 2023; Yao et al., 2023). However, as the global rainfall heterogeneity gradually increases, the degree, duration and frequency of regional drought occurrence will become more severe (IPCC, 2013; Vicente-Serrano et al., 2020), inevitably posing a serious threat to existing forest ecosystems. In arid and semi-arid regions, the growth of poplar is severely inhibited owing to water shortage, thus limiting the full economic and ecological benefits of this species (Liu et al., 2015). Liang et al. (2019) revealed that the peroxidase, catalase and superoxide dismutase activities in leaves of *Populus*×*euramericana* ‘Neva’ initially increased and then decreased as the relative soil moisture content decreased under drought stress. Yang et al. (2016) found that with the continuation of drought stress, the relative permeability of the leaf plasma membranes of the three poplar varieties increased, whereas the total soluble protein and free proline levels in leaves decreased, and the soluble sugar content remained at a higher level. Han et al. (2021) indicated that indoleacetic acid (IAA), zeatinriboside (ZR), and IAA/abscisic acid (ABA), and ZR/ABA ratios in heterogeneous leaves of *Populus euphratica* Oliv. were negatively correlated with leaf thickness, and that *P. euphratica* regulated its drought resistance at different developmental stages, adapting to an arid environment by altering the contents and ratio of these four hormones in heterogeneous leaves, as well as triggering some synergistic changes in anatomical structures. Karimi et al. (2022) found that under moderate drought-stress conditions (60% field capacity), the relative water and carotenoid contents in leaves of black poplar (*Populus nigra* L.) seedlings decrease dramatically, whereas their survival decreases by 65% under severe drought stress (30% field capacity). In addition, soil enzymes are the driving forces of soil organic matter decomposition and nutrient transformation and cycling, and they are important indicators of soil quality and ecological stability (Chen et al., 2022; Guan, 1986; Zhang et al., 2023). Bogati and Walczak (2022) showed that drought-stress conditions destroyed the microbial structure, decreased microbial activity, including enzyme production (e.g., phosphatases, catalases, oxidoreductases, ureases and hydrolases) and nutrient cycling, resulting in a decrease in soil fertility followed by lower plant productivity and economic loss. Therefore, how to enhance the drought adaptability of poplar is of great significance for the further popularization of poplar plantations and the efficient utilization of its potential economic and ecological benefits.

Super-absorbent polymers (SAP) are polymer materials with high water retention and soil improvement effects that have been applied in arid farming production in recent years. Their use can significantly increase soil moisture (Gao et al., 2022), effectively reduce nutrient leaching (Malik et al., 2023), and they can provide the advantages of water retention and fertilizer preservation. Additionally, SAP can improve the activities of soil ureases, catalases and sucrases, which aids in the cycling and transformation of nitrogen and carbon (Zhang et al., 2023). At present, the use of SAP in agriculture and forestry production is still focused on single application and combined applications with chemical or organic fertilizers (Ryan et al., 2018; Shirani Rad et al., 2013). However, the single application of a SAP has only a single function, and the combined applications of chemical fertilizers or organic fertilizers have the problems of high inputs and low utilization rates, resulting in soil hardening and environmental pollution (Guo and Wang, 2021; He et al., 2022). To ensure environmental safety and the sustainable development of agriculture and forestry, the use of plant growth-promoting rhizobacteria (PGPR) has gained attention. PGPR refers to the beneficial bacteria surviving in the rhizosphere ranges of plants that have promotional effects on plant growth or antagonistic effects on pathogenic bacteria (Goswami et al., 2016; Verma et al., 2010). The inoculation of PGPR into drought-stressed habitats can improve their drought tolerance (Bouremani et al., 2023; Chieb and Gachomo, 2023; Harkhani and Sharma, 2024). Arkhipova et al. (2005) founded that cytokinin-producing *Bacillus subtilis* promoted lettuce development under drought conditions. Arzanesh et al. (2011) reported that inoculating wheat seedlings with *Azospirillum lipoferum* strains increased leaf water content and improved root development during drought stress. Additionally, the inoculation of drought-stressed walnut seedling habitats with two different PGPR has certain effects on the contents of reduced ascorbic acid (AsA), didehydroascorbic acid (DHA), reduced glutathione (GSH), and oxidized glutathione (GSSG) (Jing et al., 2023). PGPR can also influence osmoregulation ability by raising the contents of soluble sugar, protein and proline, which subsequently enhances water absorption and growth of plants under stress conditions (Asghari et al., 2020; Rashid et al., 2022). Moreover, under drought stress conditions, PGPR facilitate plant growth by means of the production and modification of plant growth regulators and phytohormones (Barnawal et al., 2017; Khan et al., 2018). Zhang et al. (2020b) reported an increase in growth parameters and IAA accumulation in Jujube (*Ziziphus jujuba* L.) plants inoculated with *P. lini* DT6 and *Serratia plymuthica* DT8. Compared with traditional chemical and organic fertilizers, PGPR mainly aims to improve the soil microbial environment and enhance soil biological activity levels, but its application effect is restricted by environmental conditions such as soil moisture (Song et al., 2018). However, the combined application of SAP plus PGPR for afforestation in arid and semi-arid areas is helpful owing to their complementary functions. There have been some reports on the combined application of SAP and microbial agents (Song et al., 2018; Li et al., 2021; Tian et al., 2020), but little is known of the outcomes of their application in poplar, especially their effects on leaf physiological characteristics and enzyme activities in rhizosphere soil.

Thus, the current study took the limitations of independent SAP and PGPR applications as the entry point. The effects of SAP and PGPR combined applications on the physiological characteristics of leaves and enzyme activities in the rhizosphere soil of poplar seedlings under drought-stress conditions were analyzed in a pot experiment. We hypothesized that the combination of SAP and PGPR would improve the physiological characteristics and enzyme activity in rhizosphere soil of poplar seedlings. The aims were to provide an effective water-retaining and fertilization measure for poplar plantation cultivation in arid and semi-arid areas, as well as to form a theoretical basis for guiding afforestation in such regions.

## 2 Materials and methods

### 2.1 Bacterial selection and identification

The bacterial isolate F1 was isolated and purified from the *Robinia pseudoacacia* rhizosphere, and preserved in the China General Microbiological Culture Collection Center (CGMCC No. 26111, Beijing, China).

Based on 16S rRNA sequencing data and comparison of its biochemical characteristics (**Table 1**) to those described in Bergey’s Manual of Determinative Bacteriology (Holt et al., 1994), the bacterial isolate was identified as *Priestia megaterium* (previously known as *Bacillus megaterium*). The 16S RNA gene sequence of this isolate is provided in **Supplementary File 1**.

**Table 1:** Biochemical characteristics of the isolated *Priestia megaterium* F1.

L-Aspartic acid arylaminase	Phenylalanine arylaminase	α-Galactosidase	D- Galactose	Glycogen	Maltotriose	L- Rhamnose	D- ribose	D- glucose
+ ^a^	+	+	+	+	+	−	+	+

a +: positive; −,negative.

### 2.2 Basic Soil and Plant Materials

The trial was carried out from April to September 2023 in the plant nursery of Shandong Academy of Forestry, Taian (36°23′ N, 117°27′ E), Shandong Province, China.

The experimental soil was loam, with the following basic physical and chemical properties: nitrate nitrogen 1.34 mg/L, ammonium nitrogen 1.25 mg/L, available phosphorus 35.0 mg/kg, available potassium 94.1 mg/kg, organic matter 17.11 g/kg, and pH 6.54. The poplar cuttings were of *Populus* × *canadensis* ‘Huaxiong 1’ with a scion length of 15~16 cm, stem diameter of 1.7~1.9 cm, and weight of 21~23 g.

The super absorbent polymers used in the experiment was purchased from Shandong Huawei Bentonite Co., Ltd., and were detailed in **Table 2**.

**Table 2:** Selected characteristics of super absorbent polymers used in the study.

Appearance	Moisture content (%)	Particle size range (mm)	Water absorbency (g·g^-1^)	Repeat water absorbency (5 times) (g·g^-1^)	Saline (0.9%NaCl) absorbency (g·g^-1^)
Uniform particle	0.1	0.18—2.00	310	98	44

### 2.3 Experimental design

A pot experiment was performed. The pots, each with a height of 25 cm, an outer basin-mouth diameter of 29 cm, and an outer basin-bottom diameter of 19 cm, were purchased from a market. The pots were filled with 10.5 kg of soil on April 14, 2023, and the poplar cuttings were taken and planted, one per pot, on the same day. There were five treatments in the experiment, each with 10 pots: 1) CK, normal watering; 2) DR, drought stress; 3) DR+SAP, drought stress + super absorbent polymers; 4) DR+PGPR, drought stress + PGPR; and 5) DR+S+P, drought stress + super absorbent polymers + PGPR. The amount of SAP in the DR+SAP and DR+S+P treatments was 0.2% of soil quantity (21 g/pot), which was thoroughly mixed with the soil. The DR+PGPR and DR+S+P treatments were inoculated three times on July 8, 2023, July 21, 2023 and July 29, 2023, with 20 mL (approximately 1×10^9^ CFU/mL) of bacterial solution (*P. megaterium* F1) that was diluted into 1000 mL water each time and poured uniformly onto the roots of the poplar seedlings. All the plants received conventional fertilizer (N, P_2_O_5_, and K_2_O were applied at rates of 3.55, 1.94, and 3.94 g/pot, being provided by urea, superphosphate and potassium sulfate, respectively), which was mixed well with the soil at the time of potting and then applied once as a base fertilizer. After thorough watering on July 29, 2023, treatments were no longer watered, except CK, and the natural drought stress test continued until severe leaf wilting was observed (early September, 2023).

### 2.4 Data Collection and Determinations

At the end of the drought stress test (early September 2023), the aboveground and belowground parts of the seedlings were harvested and analyzed separately. Twelve leaflets at the same position on each seedling per treatment were collected, frozen with liquid nitrogen, and then stored at −60°C for determination of non-enzymatic antioxidants and other ecophysiological indicators. In addition, the rhizosphere soil was collected following the method of Wang and Zabowski (1998) for the determination of soil enzyme activity.

Physiological indexes of poplar seedlings leaves, involving the contents of AsA, DHA, GSH, GSSG, soluble sugar, soluble protein, proline, IAA, GA, ZT and ABA, and the enzyme activities of sucrase, alkaline phosphatase, catalase and urease in rhizosphere soil, were determined using specific assay kits: AsA ELISA kit (MM-35961O2), DHA ELISA kit (MM-1854O2), GSH ELISA kit (MM-33702O2), GSSG ELISA kit (MM-36072O2), soluble sugar assay kit (ADS-W-TDX039-50), soluble protein assay kit (ADS-F-SP001), proline ELISA kit (MM-33716O2), IAA ELISA kit (MM-0953O2), GA ELISA kit (MM-0125O2), ZT ELISA kit (MM-33726O2), ABA ELISA kit (MM-1185O2), sucrase ELISA kit (MM-1640O2), alkaline phosphatase ELISA kit (MM-1735O2), catalase ELISA kit (MM-1150O2), and urease ELISA kit (MM-1639O2), in which the kits used for the determination of soluble sugar and soluble protein contents were produced by Jiangsu Aidisheng Biotechnology Co., Ltd., and the kits used for the determination of the contents of proline, AsA, DHA, GSH, GSSG, IAA, GA, ZT and ABA, and the enzyme activities of sucrase, alkaline phosphatase, catalase and urease were produced by Jiangsu Meimian Industrial Co., Ltd. All the assays were performed by Standard Sci-tech Innovation (Qingdao) Pharmaceutical Technology Co., Ltd. in accordance with the manufacturer’s instructions.

### 2.5 Statistical analyses

The experiments were performed using a completely randomized design. The data were compared statistically among the five different treatments using analysis of variance (ANOVA) in SPSS (version 25.0) and least significant difference (LSD) as *post hoc* tests to distinguish the treatment groups. Differences were statistically significant at (*P*<0.05). Four principal components were derived from principal component analysis (PCA). All data are expressed as means ± standard deviation (SD) of three replicates.

## 3 Results

### 3.1 Non-enzymatic antioxidants in leaves

The antioxidant content of poplar seedling leaves showed different patterns of change in response to treatments (**Table 3**). There was no significant difference in AsA content among the treatment groups. The DHA, GSH and GSSG contents in the DR group were significantly lower than in the other treatment groups (19.08%, 14.18% and 16.94% compared with the CK, respectively), but higher in the DR+S+P group compared with the DR group (29.63%, 15.13% and 23.03%, respectively), as were those in the DR+SAP and DR+ PGPR groups. In addition, the DHA, GSH and GSSG contents in the DR+S+P group were higher than in the DR+SAP and DR+PGPR groups. Thus, drought stress significantly reduced the antioxidant contents in poplar seedling leaves, whereas the addition of PGPR or SAP to this habitat increased the antioxidant contents in poplar seedling leaves to different degrees, with the effects of adding the combination of PGPR and SAP at the same time being the most obvious.

**Table 3:** Effects of different treatments on non-enzymatic antioxidant contents in poplar seedling leaves (ng·g^-1^).

Treatment	AsA	DHA	GSH	GSSG
CK	4.06 ± 0.25a	4.56 ± 0.16ab	1.39 ± 0.06a	0.61 ± 0.02a
DR	3.58 ± 0.34a	3.69 ± 0.22c	1.19 ± 0.05c	0.51 ± 0.03b
DR+SAP	3.81 ± 0.21a	4.27 ± 0.16b	1.31 ± 0.05ab	0.58 ± 0.02a
DR+PGPR	3.88 ± 0.31a	4.44 ± 0.22ab	1.25 ± 0.04bc	0.57 ± 0.03a
DR+S+P	3.90 ± 0.20a	4.78 ± 0.15a	1.37 ± 0.02a	0.62 ± 0.05a

Data are mean ± SD. CK, normal watering; DR, drought stress; DR+SAP, drought stress + super absorbent polymers; DR+PGPR, drought stress + PGPR; DR+S+P, drought stress + super absorbent polymers + PGPR; AsA, reduced ascorbic acid; DHA, dehydroascorbate; GSH, reduced glutathione; GSSG, oxidized glutathione. Different letters in the same column indicate significant differences among treatments at *P*<0.05 by LSD.

### 3.2 Osmoregulatory substances in leaves

**Figure 1:**
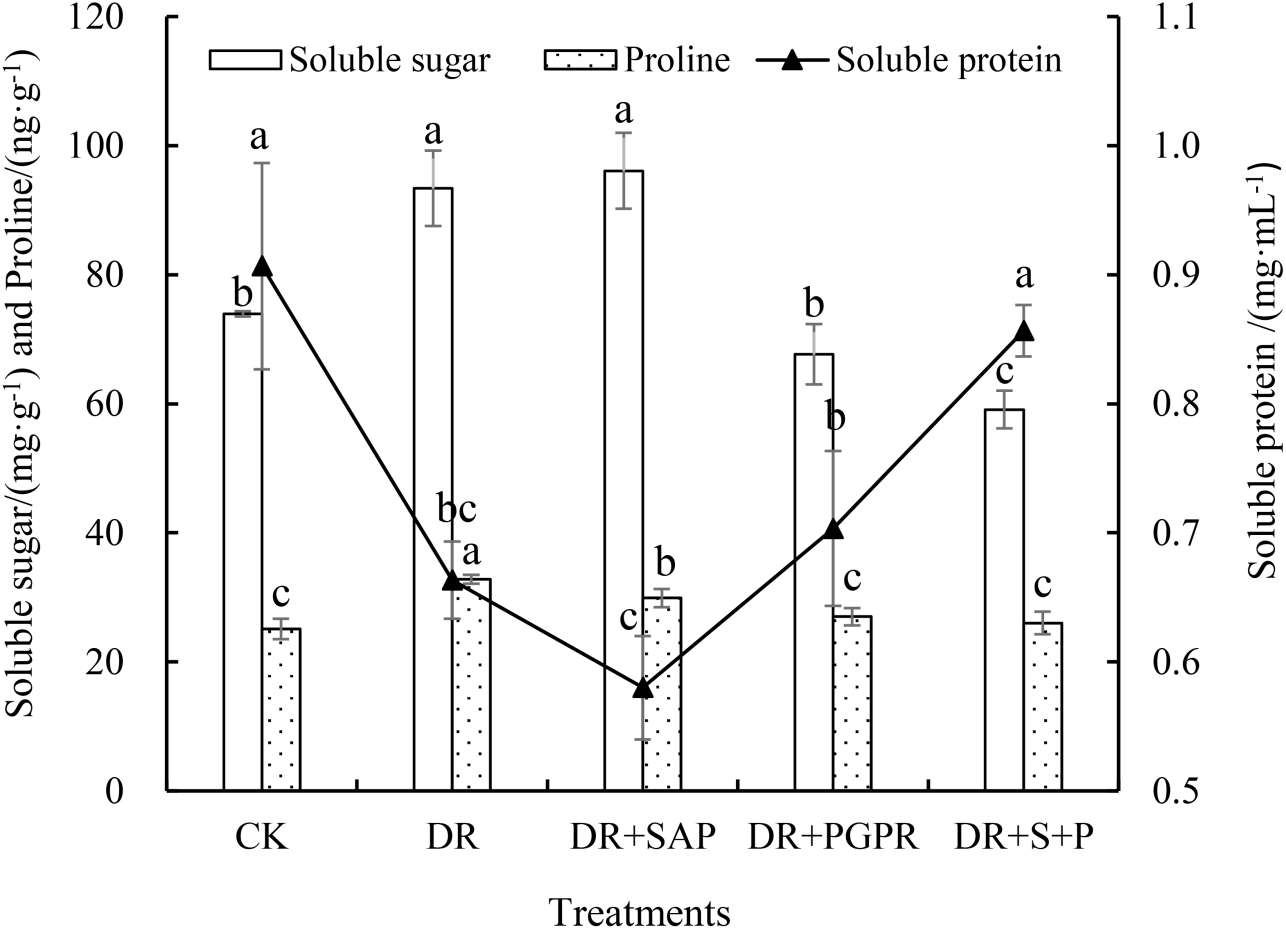
Effects of different treatments on the osmoregulatory substance contents in poplar seedling leaves. Bars are means, and error bars are standard deviations. CK, normal watering; DR, drought stress; DR+SAP, drought stress + super absorbent polymers; DR+PGPR, drought stress + PGPR; DR+S+P, drought stress + super absorbent polymers + PGPR; Different letters in the same column indicate significant differences among treatments at *P*<0.05 by LSD.

The osmoregulatory substance contents of poplar seedling leaves differed significantly among treatment groups (**Figure 1**). The soluble protein content in the DR group was 26.84% lower than that of the CK, where that in the DR+S+P was not significantly different from that of the CK, but was significantly higher than that of the DR group (by 29.15%).In addition, the soluble sugar and proline contents in the DR group were significantly higher, by 26.28% and 30.55%, respectively, in comparison with the CK. The soluble sugar contents of the DR+PGPR and DR+S+P groups were significantly lower than that of the DR group by 27.53% and 36.73%, respectively. The proline content of the DR+SAP, DR+PGPR and DR+S+P groups were also obviously lower than that of the DR group, with decreases of 8.85%, 17.63% and 20.66%, respectively. The soluble sugar and proline contents of the DR+S+P group were the lowest among the observed values of drought stress treatments. Thus, drought stress reduced the soluble protein content in poplar seedlings leaves, but it promoted the accumulation of soluble sugars and proline in leaves. The simultaneous addition of SAP and PGPR under drought stress significantly increased the soluble protein content in poplar seedling leaves and significantly reduced the proline and soluble sugar contents in leaves.

### 3.3 Leaf endogenous hormones

The facilitatory hormones IAA, GA and ZT promote plant growth and development, whereas the stress hormone ABA inhibits plant growth. The contents of IAA, GA and ZT of poplar seedling leaves in the DR group were significantly lower than those in the CK, with decreases of 9.47%, 11.13% and 12.83%, respectively (**Table 4**). The IAA and ZT contents in the DR+SAP and DR+PGPR groups were higher than those in the DR group, but not significantly so. The GA content in the DR+PGPR group was markedly higher than that in the DR group. Additionally, the IAA and GA contents in the DR+S+P group increased significantly by 12.55% and 11.73%, respectively, compared with the DR group. The ABA level in each treatment group followed the order: DR > DR+SAP ≈ DR+PGPR > DR+S+P ≈ CK, with the ABA content of the DR group being significantly higher than in the other treatment groups (**Table 4**). The ABA levels in the DR+SAP, DR+PGPR and DR+S+P groups were reduced significantly, by 11.60%, 15.06%, and 23.40%, respectively, compared with the DR group, with that in the DR+S+P group being markedly lower than in the DR+SAP and DR+PGPR groups. Thus, drought stress reduced the IAA, GA and ZT contents in poplar seedling leaves but increased the ABA content, whereas the simultaneous addition of SAP and PGPR under drought stress significantly promoted the accumulation of IAA and GA in poplar seedling leaves and significantly reduced the ABA content.

**Table 4:** Effects of different treatments on endogenous hormone contents in poplar seedling leaves.

Treatment	IAA/(μg·g^-1^)	GA/(pg·mg^-1^)	ZT/(ng·g^-1^)	ABA/(μg·g^-1^)
CK	0.88 ± 0.04a	4.70 ± 0.10a	3.43 ± 0.17a	2.36 ± 0.03c
DR	0.80 ± 0.07b	4.18 ± 0.03b	2.99 ± 0.15b	3.28 ± 0.10a
DR+SAP	0.83 ± 0.03ab	4.08 ± 0.05b	3.02 ± 0.11b	2.90 ± 0.09b
DR+PGPR	0.82 ± 0.02ab	4.50 ± 0.20a	3.28 ± 0.15ab	2.78 ± 0.22b
DR+S+P	0.90 ± 0.03a	4.67 ± 0.21a	3.19 ± 0.18ab	2.51 ± 0.10c

Data are mean ± SD. CK, normal watering; DR, drought stress; DR+SAP, drought stress + super absorbent polymers; DR+PGPR, drought stress + PGPR; DR+S+P, drought stress + super absorbent polymers + PGPR; IAA, indoleacetic acid; GA, gibberellin; ZT, zeatin; ABA, abscisic acid. Different letters in the same column indicate significant differences among treatments at *P*<0.05 by LSD.

### 3.4 Leaf endogenous hormone balance

**Figure 2:**
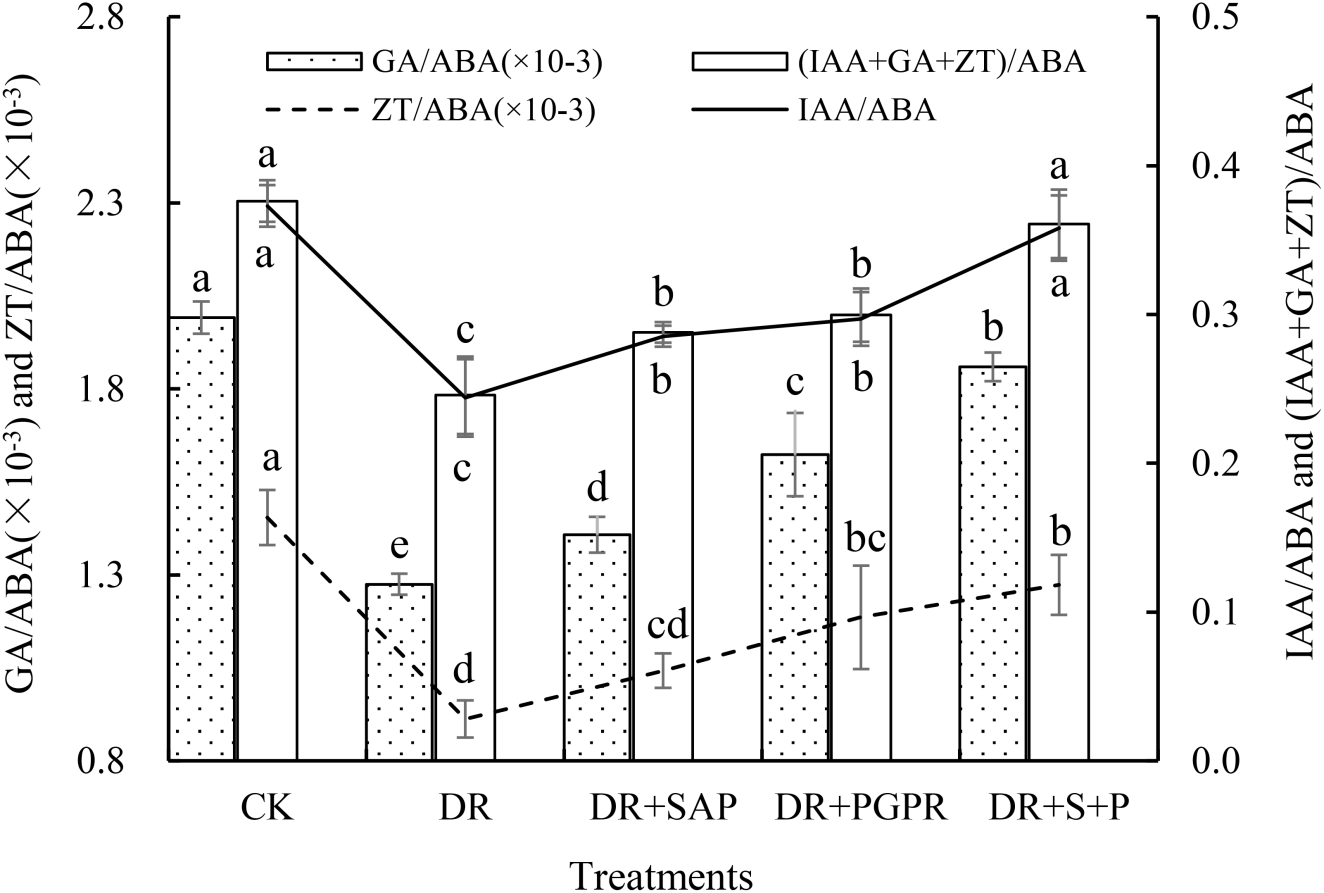
Effects of different treatments on the endogenous growth-promoting hormone to ABA ratios in poplar seedling leaves. Bars are means, and error bars are standard deviations. CK, normal watering; DR, drought stress; DR+SAP, drought stress + super absorbent polymers; DR+PGPR, drought stress + PGPR; DR+S+P, drought stress + super absorbent polymers + PGPR; IAA, indoleacetic acid; GA, gibberellin; ZT, zeatin; ABA, abscisic acid. Different letters in the same column indicate significant differences among treatments at *P*<0.05 by LSD.

The regulation of plant growth and development by endogenous hormones is often the result of the combined actions of multiple hormones, which depend not only on the concentrations of the hormones themselves, but also on the appropriate ratio and balance among the hormones (Xing et al., 2022). The IAA/ABA and (IAA+GA+ZT)/ABA ratios in poplar seedling leaves showed basically consistent patterns of change (**Figure 2**). The IAA/ABA and (IAA+GA+ZT)/ABA ratios in the DR group were the lowest and were significantly lower than those in the other groups, including being 34.56% and 34.67% lower than those of the CK, respectively. The IAA/ABA and (IAA+GA+ZT)/ABA ratios in the DR+S+P group were not markedly different from those of the CK, but they were significantly higher than those in the DR, DR+SAP and DR+PGPR groups. The IAA/ABA ratios were significantly increased by 46.72%, 25.44% and 20.54%, and the (IAA+GA+ZT)/ABA ratios were significantly increased by 46.85%, 25.36% and 20.48%, respectively, compared with those of the DR, DR+SAP and DR+PGPR groups. The order of the GA/ABA ratio among treatment groups was CK > DR+S+P > DR+PGPR > DR+SAP > DR, with significant differences among all groups. Additionally, the CK had the largest ZT/ABA ratio, followed by the DR+S+P and DR+PGPR groups, all of which were significantly higher than that of the DR group. Thus, drought stress significantly reduced the IAA/ABA, GA/ABA, ZT/ABA and (IAA+GA+ZT)/ABA ratios of poplar seedling leaves, whereas the addition of SAP or PGPR significantly increased these ratios under drought stress, with the increase being especially prominent after the simultaneous addition of SAP and PGPR.

### 3.5 Enzyme activity in rhizosphere soil

The urease and sucrase activities in the rhizosphere soil of poplar seedlings varied among treatment groups (**Table 5**). The urease activity in the DR group was 16.34% lower than that of the CK, and the difference was at a significant level. The urease activities in the DR+SAP and DR+S+P groups were significantly higher, by 12.04% and 14.37%, respectively, compared with the DR group. Additionally, the sucrase activity of the DRgroup significantly decreased by 15.58% in comparison to the CK. The sucrase activities in the DR+PGPR and DR+S+P treatments increased compared with the DR group, but not significantly so. The catalase activity was reduced in the DR group in comparison to the CK, whereas the catalase activity was somewhat increased in the DR+S+P group compared with the DR group. However, the differences among these three groups were not significant. Moreover, the alkaline phosphatase activity did not show significant differences among treatment groups. Thus, drought stress reduced the activities of urease, sucrase and catalase in the rhizosphere soil of poplar seedlings, whereas the simultaneous addition of SAP and PGPR increased their activities to different degrees. This indicates a certain promotional effect for improving the micro-domain environment in the rhizosphere soils of poplar seedlings.

**Table 5:** Effects of different treatments on enzyme activities in rhizosphere soils of poplar seedlings.

Treatment	Urease/(IU·g^-1^)	Sucrase/(U·g^-1^)	Catalase/(U·mg^-1^)	Alkaline phosphatase/(IU·g^-1^)
CK	11.12 ± 0.55a	8.64 ± 0.13a	0.0787 ± 0.0016a	0.670 ± 0.036a
DR	9.30 ± 0.73b	7.30 ± 0.42b	0.0769 ± 0.0026ab	0.670 ± 0.000a
DR+SAP	10.42 ± 0.36a	8.66 ± 0.46a	0.0811 ± 0.0046a	0.667 ± 0.025a
DR+PGPR	9.13 ± 0.12b	7.58 ± 0.24b	0.0708 ± 0.0020b	0.623 ± 0.040a
DR+S+P	10.64 ± 0.12a	7.82 ± 0.13b	0.0786 ± 0.0059a	0.627 ± 0.038a

Data are mean ± SD. CK, normal watering; DR, drought stress; DR+SAP, drought stress + super absorbent polymers; DR+PGPR, drought stress + PGPR; DR+S+P, drought stress + super absorbent polymers + PGPR. Different letters in the same column indicate significant differences among treatments at *P*<0.05 by LSD.

### 3.6 Principal component analysis

As presented in **Table 6**, four principal components were obtained based on the eigenvector greater than 1. Their contribution rates were 59.468%, 13.316%, and 7.975%, respectively, and the accumulated contribution rate was 87.327%, which could represent the overall system status. In the first principal component, ABA, IAA/ABA, GA/ABA, ZT/ABA, and (IAA + GA + ZT)/ABA had larger loads, and the correlation coefficients were -0.960, 0.977, 0.963, 0.930, and 0.977, respectively. In the second principal component, catalase, sucrase, and alkaline phosphatase had larger loads, and the correlation coefficients were 0.758, 0.746, and 0.619, respectively. In the third principal component, ZT, catalase, and AsA had larger loads, and the correlation coefficients were 0.466, -0.446, and -0.656, respectively. In the fourth principal component, GA and AsA possessed larger loads, and the correlation coefficients were 0.461 and 0.550, respectively. Principal component analysis demonstrated that the endogenous hormone balance, stress hormone, GA, ZT, and AsA in leaves, along with rhizosphere soil enzyme activities, were crucial indexes for evaluating the drought resistance of poplar seedlings.

**Table 6:** Loading matrix and contribution rate of principal component factors.

Index	PC1	PC2	PC3	PC4
(IAA+GA+ZT)/ABA	0.977	0.058	0.072	-0.072
IAA/ABA	0.977	0.059	0.070	-0.073
GA/ABA	0.963	-0.048	0.054	0.234
ABA	-0.960	-0.052	-0.018	-0.030
ZT/ABA	0.930	-0.035	0.219	-0.007
Proline	-0.884	0.143	-0.080	-0.084
DHA	0.858	-0.111	-0.336	-0.173
GSH	0.823	0.347	0.109	0.005
GSSG	0.818	0.094	-0.439	-0.281
Soluble protein	0.812	-0.127	0.182	0.360
IAA	0.768	0.038	0.127	-0.384
GA	0.767	-0.285	0.035	0.461
Soluble sugar	-0.722	0.594	0.216	-0.151
ZT	0.700	-0.218	0.466	-0.166
Urease	0.687	0.584	0.096	-0.186
Catalase	0.064	0.758	-0.446	0.205
Sucrase	0.442	0.746	0.032	-0.159
Alkaline phosphatase	-0.243	0.619	0.419	0.550
AsA	0.483	0.032	-0.656	0.349
Eigenvalue	11.299	2.530	1.515	1.248
Contribution rate/%	59.468	13.316	7.975	6.568
Accumulated contribution rate/%	59.468	72.784	80.759	87.327

IAA, indoleacetic acid; GA, gibberellin; ZT, zeatin; ABA, abscisic acid; AsA, reduced ascorbic acid; DHA, dehydroascorbate; GSH, reduced glutathione; GSSG, oxidized glutathione.

**Figure 3:**
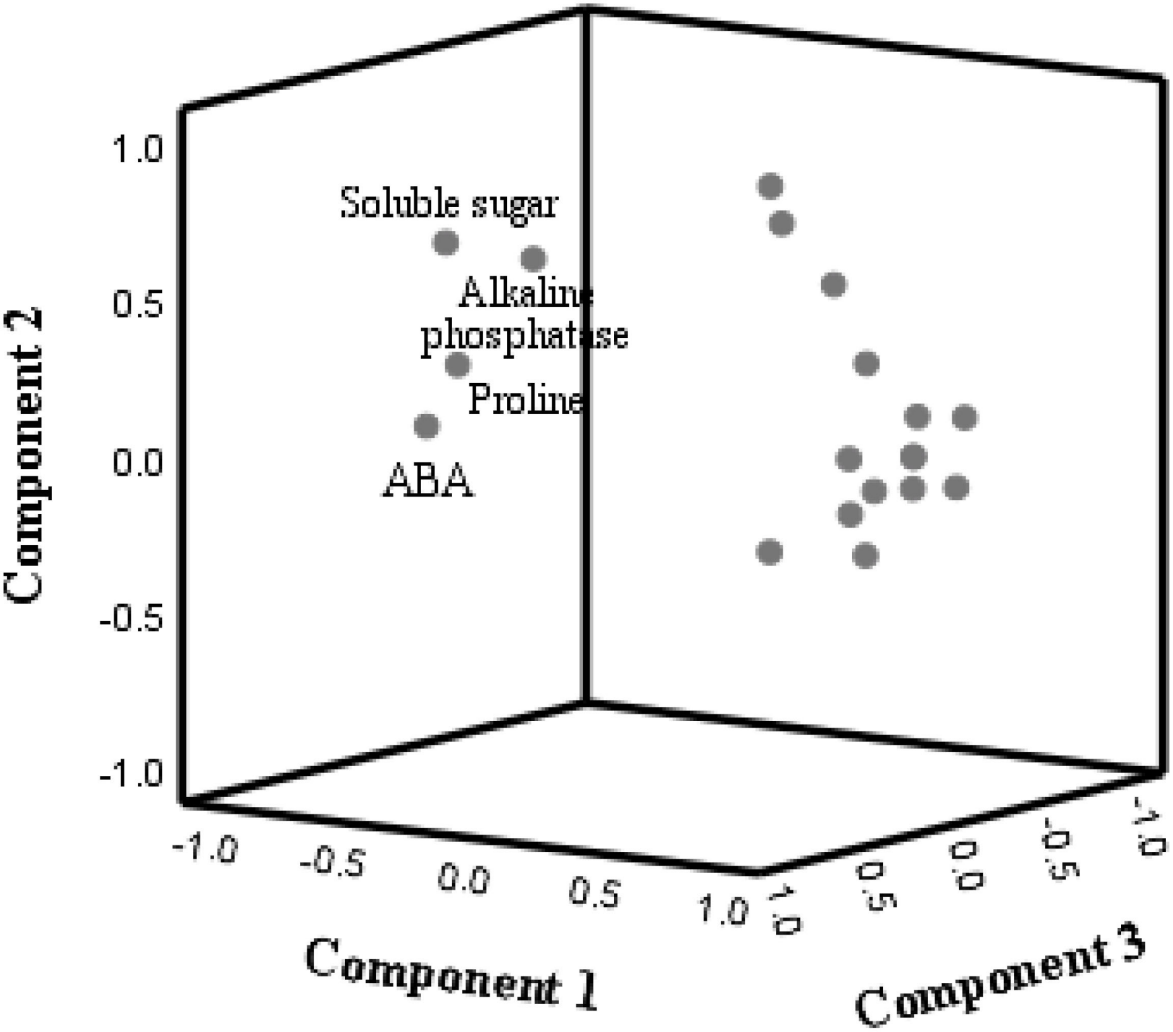
Results of principal component analysis.

Furthermore, the 19 indicators that SAP and PGPR influenced the drought resistance of poplar seedlings could be divided into two relatively independent groups (**Figure 3**). Among them, the indicators of soluble sugar, proline, ABA, and rhizosphere soil alkaline phosphatase constituted a relatively independent group. When SAP or PGPR was added under drought stress, the general alteration of these four indexes presented a downward trend. The remaining indexes formed another independent group. When SAP or PGPR was added under drought stress, the change rule was that these indexes showed an overall upward trend.

## 4 Discussion

### 4.1 SAP and PGPR increased the antioxidant contents of poplar seedlings

In arid and semi-arid regions, water is the main factor restricting the growth and development of poplar. Drought stress can disrupt the dynamic balance of reactive oxygen species (ROS) in plants, resulting in significant ROS accumulation. Under adverse conditions, AsA, DHA, GSH and GSSG are important non-enzymatic antioxidants in plants that play crucial roles in the scavenging of ROS (Jiang et al., 2022). In this study, the contents of DHA, GSH and GSSG in poplar seedling leaves were dramatically reduced under drought stress (**Table 3**), whereas Luo et al. (2022) showed that the AsA and DHA contents in hairgrass (Deschampsia caespitosa) leaves steadily increased under heavily drought. This inconsistency may result from the stronger drought tolerance of Deschampsia caespitosa compared with poplar seedlings, and it may also be related to the differences in stress intensity and stress duration. Here, the addition of SAP significantly increased the antioxidant contents in poplar seedling leaves under drought stress (**Table 3**). This may result from the special polymer chain structure of the SAP, which can better retain and store water after application in the soil. They also provide better water conditions for the growth of poplar seedlings in the short term, thus alleviating the degree of drought stress. The PGPR inoculation may also increase the antioxidant contents in poplar seedlings leaves (**Table 3**). On the one hand, the effective functional bacteria of PGPR may have formed dominant flora in the soil environments of poplar seedlings, formed a competitive relationship with pathogenic bacteria, inhibited the growth of harmful bacteria and improved the nutrient absorption of poplar seedlings; thereby, effectively alleviating the drought-related stress (Tian et al., 2022). On the other hand, the activities of microorganisms and their metabolites may have further activated soil nutrients, ameliorated soil pH and soil aeration; thereby, improving the water and fertilizer retention capacities of the soils (Omara et al., 2022). Furthermore, under drought stress, the simultaneous addition of SAP and PGPR increased the antioxidant contents of poplar seedlings the most among the treatments (**Table 3**), which may be attributed to SAP providing good water conditions for the acclimatization of PGPR to the soil and for bacterial propagation (Tian et al., 2020). This would contribute to increased microbial activity and the transformation of soil available nutrients. Moreover, the combined addition improved the rhizosphere microenvironment, promoted the absorption and utilization of mineral nutrients by the root system of poplar seedlings, and increased the production of metabolites that promote plant growth (Nihorimbere et al., 2011).

### 4.2 SAP and PGPR increased soluble protein content and decreased the soluble sugar and proline contents of poplar seedlings

When plants experience drought, high temperature or salt stress, they maintain cell turgor, prevent cell dehydration, enhance cell water-holding capacity and protect the cellular biofilm by increasing levels of small-molecule organic osmoregulatory substances (Guo et al., 2018; Khoyerdi et al., 2016). In the current study, drought stress caused significant increases in proline and soluble sugar contents in poplar seedling leaves (**Figure 1**). Similar findings were obtained by Guo et al. (2018) in *Lycium ruthenicum* Murr. seedlings. Drought stress may seriously affect the normal growth and development of poplar seedlings, resulting in the plant’s osmotic pressure continuously increasing. Consequently, to maintain the osmotic pressure balance and reduce water loss, the plant secretes large amounts of soluble sugars and free proline to regulate the osmotic pressure balance (Wang et al., 2023). By contrast, the contents of proline and soluble sugars in poplar seedlings leaves under drought stress were significantly decreased by the addition of PGPR alone or by the addition of PGPR plus SAP (**Figure 1**). It is thought that proline acts as a stress marker in addition to its role as an osmoprotectant, indicating that plants accumulate more proline as a result of biotic stress or less proline by reason of stress reduction. Hence, the lower proline accumulation in treated poplar seedlings suggested that these plants were less exposed to drought and did not require excessive proline accumulation to withstand this lower level of drought stress (Nader et al., 2024).

The present study also concluded that the soluble protein content of poplar seedling leaves significantly decreased under drought stress (**Figure 1**). However, Ferioun et al. (2023) investigated drought stress and determined that it caused a significant increase in the soluble protein content of seven Moroccan barley (*Hordeum vulgare* L.) cultivars. This may be related to drought resistance, stress intensity, stress duration and other factors, and the specific mechanisms need to be further studied. Additionally, the content of soluble protein in poplar seedling leaves increased to different degrees in response to the addition of PGPR alone or both SAP and PGPR under drought-stress conditions (**Figure 1**). Ilyas et al. (2020) also reported similar results by inoculating bacterial strains in wheat under drought stress.

### 4.3 SAP and PGPR increased the facilitatory hormone content and facilitatory hormone: stress hormone ratio, but decreased the stress hormone content of poplar seedlings

Plants under drought stress can transmit drought-related information, allowing them to regulate their growth and development through the increase or decrease of endogenous hormones to alleviate the stress (Wang et al., 2008; Wahab et al., 2022). In our study, drought stress significantly reduced the contents of IAA, GA and ZT in poplar seedlings leaves, whereas the addition of PGPR alone, or both SAP and PGPR, under drought stress significantly increased the contents of these growth-promoting hormones (**Table 4**). This suggested that inoculation with PGPR promoted root growth and enhanced root water and nutrient uptake by increasing the content of growth-promoting hormones in poplar seedling leaves, which in turn improved the plants’ drought resistance. In addition, ABA, as an important stress hormone accumulated in plants, regulates water loss by controlling stomatal closure and stress signaling pathways during water deficits. The more severe the water stress, the higher the ABA content in the plant (Zhang et al., 2020a). In this experiment, the ABA content in poplar seedling leaves under drought stress markedly increased, whereas the ABA content significantly decreased after inoculation with PGPR or the addition of SAP (**Table 4**). The greatest reduction was achieved with the simultaneous addition of SAP and PGPR (**Table 4**). The study of Zhang et al. (2020a) on *Zizyphus jujuba* showed that inoculations of *Pseudomonas koreensis*, *Bacillus filamentosus* and their hybrids effectively reduced the ABA content under different drought-stress levels. This also indirectly reflected that under stress conditions, PGPR inoculation slows down the effects of plant stress. The reticulation structure of SAP has a strong adsorption effect on soil moisture, which effectively improves soil water fixation (Bai et al., 2010), thereby contributing to the PGPR effect.

Hormone regulation is accomplished by the coordinated actions of plant endogenous hormones, and the balance among hormones is more important than the level of a single hormone (Peleg and Blumwald, 2011). Changes in the ratios between different hormones reflect the transformation of the plant’s growth center (Großkinsky et al., 2016). In this study, the ratios of IAA/ABA, GA/ABA, ZT/ABA and (IAA+GA+ZT)/ABA in leaves of poplar seedlings were markedly reduced under drought stress (**Figure 2**), with the general trend of the coordination among the four hormones resulting in stomatal closure and growth inhibition. This indicated that drought stress altered the dynamic balance among endogenous hormones in poplar seedling leaves, encouraging the reproductive process and the occurrence of premature senescence. The addition of SAP or PGPR under drought stress significantly increased these ratios, with the greatest increased amplitude being observed with the simultaneous addition of SAP and PGPR (**Figure 2**). This suggests that the addition of SAP or PGPR induces the coordination among these four endogenous hormones to encourage growth.

### 4.4 SAP and PGPR increased soil enzyme activity in the rhizosphere of poplar seedlings to different degrees

Soil enzymes, mainly derived from soil microorganisms, soil animals, plant roots and residues, are the metabolic driving force of soil organisms (Wang et al., 2022). They are involved in soil nutrient transformation and transport, which are the bioactive indicator of the soil’s ability to supply nutrients (Neemisha, 2022). In this study, the activities of urease, sucrase and catalase in rhizosphere soil of poplar seedlings decreased under drought stress, whereas the addition of both SAP and PGPR in this habitat increased their activities to different degrees (**Table 5**). This may result from SAP providing good water conditions for PGPR to acclimatize and enter the soil acclimatization environment, as well as encouraging bacterial propagation. During bacterial propagation, PGPR produce large numbers of metabolites, which improve the soil structure, and a good soil structure provides the basis for the long-lasting performance of SAP. Thus, it helps SAP to better perform its functions of water retention and storage. Moreover, while improving the rhizosphere soil microenvironment, PGPR activate the indigenous microorganisms in the soil to a certain extent, which can jointly accelerate the decomposition of organic compounds and provide substrates for enzymatic reactions. This not only increases the nutrient content of the soil and promotes the growth of microorganisms (Liu et al., 2022), but it also increases the amounts of extracellular enzymes released and the secretion of substances associated with the enhancement of soil enzyme activity (Li et al., 2020). Thus, SAP locks in water as well as nutrients, and the SAP plus PGPR combination improves soil biological properties, providing a greater level of water and nutrients for poplar seedling growth in drought-stressed environments (Tian et al., 2020). Similar conclusions were reached by Wang et al. (2023) in their study on soil enzyme activities of *Medicago sativa* L. inoculated with microbial agents under drought-stress conditions.

## 5 Conclusions

The results of the current study show that the simultaneous addition of super-absorbent polymers and *P. megaterium* under drought stress increased the contents of non-enzymatic antioxidants, soluble proteins, facilitatory hormones, and the facilitatory hormone: stress hormone ratio in leaves. It also significantly decreased the soluble sugar, proline and stress hormone contents. Thus, the simultaneous addition of super-absorbent polymers and *P. megaterium* under drought-stress conditions enhanced the drought adaptive capacities of poplar seedlings mainly by means of regulating the non-enzymatic antioxidant, osmotic regulator, and endogenous hormone contents and the endogenous hormone balance in poplar seedling leaves. These results indicate that the combined utilization of super absorbent polymers and PGPR can not only enhance the adaptability of poplar seedlings to drought resistance, but also decrease the application of fertilizer and facilitate the development of water-saving agroforestry, thereby contributing to the green and sustainable development of agroforestry production. Consequently, it offers a new technical approach for agroforestry production in arid and semi-arid areas worldwide. Furthermore, the effects of super-absorbent polymers and PGPR on drought resistance and adaptability of poplar plantations in drought-affected areas require further study and long-term observation.

## Data Availability

The original contributions presented in the study are included in the article/**Supplementary Material**. Further inquiries can be directed to the corresponding author/s.

## Supplementary material

Supplementary File 1 The 16S RNA gene sequence of *P. megaterium* F1 used in our study.
